# Validation of a vitamin D replacement strategy in vitamin D-insufficient patients with lymphoma or chronic lymphocytic leukemia

**DOI:** 10.1038/bcj.2017.9

**Published:** 2017-02-03

**Authors:** J G Sfeir, M T Drake, B R LaPlant, M J Maurer, B K Link, T J Berndt, T D Shanafelt, J R Cerhan, T M Habermann, A L Feldman, T Witzig

**Affiliations:** 1 Division of Endocrinology, Diabetes, Metabolism and Nutrition Department of Internal Medicine Mayo Clinic, Rochester, MN, USA; 2 Division of Biomedical Statistics and Informatics Mayo Clinic, Rochester, MN, USA; 3 Holden Comprehensive Cancer Center University of Iowa, Iowa City, IA, USA; 4 Mayo Clinic Health Science Research, Rochester, MN, USA; 5 Mayo Clinic Cancer Center Mayo Clinic, Rochester, MN, USA; 6 Department of Laboratory Medicine and Pathology Mayo Clinic, Rochester, MN, USA

Circulating serum 25-hydroxyvitamin D (25(OH)D) levels reflect the net contribution of food and supplement intake as well as skin production through sun exposure, and are widely accepted as the standard to assess for individual adequacy or insufficiency.^1^ Recent work has demonstrated the pleiotropic effects of vitamin D on cellular differentiation, proliferation, apoptosis and angiogenesis.^2^

Vitamin D insufficiency has been linked to poor prognosis in patients with malignancy, particularly lymphoid malignancies.^3, 4^ Previous studies from our group, subsequently validated by others, demonstrate that insufficient vitamin D levels are independent, adverse prognostic factors for time to therapy and overall survival in patients with chronic lymphocytic leukemia (CLL) and event-free survival/overall survival in patients with diffuse large B-cell lymphoma and peripheral T-cell lymphoma.^5, 6, 7^ More recently, vitamin D insufficiency has been shown to be an adverse prognostic factor for follicular lymphoma.^8^

To date, however, there has been no clear evidence as to whether vitamin D replacement in the setting of hypovitaminosis D has an impact on prognosis in patients with lymphoid malignancies. This key question is currently being addressed in our ongoing clinical trial undertaken by the University of Iowa/Mayo Clinic Lymphoma SPORE with collaboration from Emory University (clinicaltrials.gov #NCT01787409). Our overall hypothesis is that survival can be improved in patients with vitamin D insufficiency following vitamin D replacement to a target 25(OH)D level of ⩾30 ng/ml. In this first report from the ongoing trial, we evaluated whether our vitamin D replacement strategy for insufficient patients was effective at achieving 25(OH)D levels ⩾30 ng/ml prior to entry into a follow-up period of 3 years, during which these levels are maintained.

The study protocol has been reviewed and approved by the Institutional Review Boards at each study site, with written informed consent obtained from all participants. Study eligibility criteria are adults with (a) newly diagnosed untreated diffuse large B-cell lymphoma scheduled for treatment with six cycles of R-CHOP or a similar regimen; (b) untreated peripheral T-cell lymphoma requiring combination chemotherapy; or (c) Rai stage 0 or 1 CLL who are asymptomatic and candidates for observation. All vitamin D measurements were made by liquid chromatography-tandem mass spectrometry at the Mayo Medical Laboratories. Vitamin D insufficiency was defined as a serum 25(OH)D level <25 ng/ml. This value reflects a value midway between the optimal levels of 20 ng/ml proposed by the Institute of Medicine and 30 ng/ml recommended by the Endocrine Society.^1, 9^ The goal is to achieve a blood level ⩾30 ng/ml based on our previous data showing association with improved event-free survival/overall survival.

Vitamin D replacement in all insufficient patients is divided into a loading phase (described in this letter) and a maintenance phase, using cholecalciferol (vitamin D3) 50 000 International Units (IU) capsules, supplied complimentary by Bio-Tech Pharmacal (Fayetteville, Arkansas). Insufficient patients are administered one 50 000 IU capsule weekly for 12 weeks followed by a repeat 25(OH)D blood level. If the blood level is <30 ng/ml and the patient has been compliant with the dosing, the dose is increased to 50 000 IU twice weekly for an additional 12 weeks followed by another 25(OH)D blood level. Once the goal of ⩾30 ng/ml has been achieved, the patients proceed to maintenance phase and receive 50 000 IU monthly for the remainder of the 3-year active treatment period.

The primary study goal is to determine if vitamin D replacement in insufficient patients can improve 12-month event-free survival in the lymphoma arm, and increase time to chemotherapy and overall survival in the CLL arm. Since the study goals hinge on achievement of 25(OH)D levels of ⩾30 ng/ml, we demonstrate in this initial report that our vitamin D replacement strategy, which is simple to implement for physicians and patients, is safe and effective.

Between January 2013 and June 2016, 158 patients were enrolled into the study and completed at least 12 weeks of follow-up. One-hundred forty-five (92%) patients had a diagnosis of lymphoma (combined diffuse large B-cell lymphoma and T-cell NHL), and 13 had CLL. Seventy-one (45%) patients had vitamin D insufficiency at baseline with a mean 25(OH)D of 17.0±5.2 ng/ml (Table 1). Seventy patients completed 12 weeks of vitamin D replacement and one patient elected not to take the replacement dose. At the end of the 12-week induction period, 97% (69/70) of insufficient patients had achieved vitamin D sufficiency, with a new mean 25(OH)D of 54.7±13.9 ng/ml. The one patient in whom 25(OH)D levels remained below 30 ng/ml at 12 weeks had severe hypovitaminosis D at study entry (baseline level, 6.0 ng/ml), and had achieved a 25(OH)D level at 12 weeks of 28 ng/ml. After an additional 12 weeks of vitamin D at 50 000 IU twice weekly, this patient achieved target 25(OH)D levels (36 ng/ml). The remaining 87 (55%) patients had baseline 25(OH)D levels ⩾25 ng/ml with a mean of 36.6±9.6 ng/ml, with 22 subjects having 25(OH)D levels ⩾25 but <30 ng/ml at baseline. As per study protocol, these subjects were not offered any vitamin D replacement or supplementation. At the 12-week follow-up visit, 25(OH)D levels in this cohort demonstrated a mean of 34.4±10.7 ng/ml. Of note, slightly more than one-third of these patients (37.9%) had a 25(OH)D level ⩽30 ng/ml at 12 weeks and 16% had a 25(OH)D level ⩽25 ng/ml.

Baseline and follow-up total serum calcium were documented on 92% of the entire cohort. At 12 weeks, there was no significant change in total serum calcium levels from baseline in either group (Table 2). Eight patients had serum calcium values above the upper normal limit (10.1 mg/dl at Mayo Medical Laboratories) at baseline (median 10.65 mg/dl, range 10.2–11.2 mg/dl), and nine patients had serum calcium values above the upper normal limit at 12 weeks (median 10.4 mg/dl, range 10.2–12.6). One patient in the vitamin D replacement cohort had a significant but transient elevation in serum calcium to 12.6 mg/dl. However, this elevation occurred in the setting of an antibiotic-associated acute kidney injury that resolved within 1 week, with the serum calcium returning to the normal range at 9.3 mg/dl. The patient remained on study and subsequent serum calcium values were 9.8  and 9.4 mg/dl at 24 and 36 weeks, respectively. There were no reported cases of nephrolithiasis.

Our replacement strategy for vitamin D in patients with hypovitaminosis D largely follows the recommendations for the general population from the Endocrine Society.^9^ No recommendations exist that are specific to patients with malignancies. Although previous studies have looked at cancer incidence with vitamin D supplementation or replacement,^10, 11, 12^ we are not aware of any studies evaluating vitamin D replacement strategies in patients with lymphoid malignancies. Two recent studies evaluated vitamin D replacement strategies in patients with solid tumors and showed suboptimal results. Daily treatment with 20 000 IU of vitamin D for 14 days followed by 10 000 IU for 7 days in 80 patients with advanced lung cancer failed to achieve target 25(OH)D levels at 21 days in 44% of patients, including 1% that remained below 10 ng/ml.^13^ In another recent trial, only 30% of patients with early breast cancer and hypovitaminosis D had normal 25(OH)D levels after 6 months of tailored replacement versus 13% of those receiving daily supplementation.^14^

As shown here, cholecalciferol 50 000 IU once weekly for 12 weeks resulted in the successful achievement of 25(OH)D sufficiency in 97% of patients. In only one patient, 24 weeks of replacement rather than 12 weeks was needed to achieve the target 25(OH)D level of 30 ng/ml. In that patient, the hypovitaminosis D severity at baseline was the likely etiology of the time needed to achieve sufficiency.

The results from this ongoing vitamin D replacement study in patients with lymphoma or CLL and vitamin D insufficiency, including those undergoing induction chemotherapy, provide evidence that vitamin D can be safely and efficiently replaced with achievement of target 25(OH)D levels of 30 ng/ml at 12 weeks without toxicity. We did not find any significant change in total serum calcium levels after 12 weeks of vitamin D replacement (Table 2), and no patients experienced nephrolithiasis. Although mild hypercalcemia was documented in several patients in the study, our protocol was not designed to investigate the etiology of hypercalcemia. Clinically, hemoconcentration may cause mild hypercalcemia during fasting venipuncture.^15^ In the few patients in whom mild hypercalcemia was noted, mild fasting associated dehydration was the most likely explanation as no patient was determined to have persistent hypercalcemia. In fact, the number of patients in our cohort with serum calcium values above 10.1 mg/dl declined during longitudinal evaluation: eight patients at 6 months and one patient at 12 months.

Finally, we suggest that this information on vitamin D dosing should also be applicable to other patients with malignancies and vitamin D insufficiency. Compared to published strategies of vitamin D replacement in this population, we offer a simple, effective and safe strategy that aligns with published guidelines in the general population. Patients should be counseled that vitamin D replacement appears to be safe; however, whether it actually alters cancer prognosis remains to be determined.

## Figures and Tables

**Table 1:** Vitamin D levels at baseline and after 12 weeks

	*Vitamin D-insufficient group*		*Vitamin D-sufficient group*	
	*25(OH)D at baseline (ng/ml)*	*25(OH)D at 12 weeks (ng/ml)*	*25(OH)D at baseline (ng/ml)*	*25(OH)D at 12 weeks (ng/ml)*
*N*	71	71	87	87
Mean±s.d.	17.0±5.2	54.7±13.9	36.6±9.6	34.4±10.7
Median	18.0	53.0	35.0	33.0
Range	6.0–24.0	28.0–95.0	25.0–74.0	10.0–82.0
⩽25 ng/ml	71	1	0	14

Abbreviation: 25(OH)D, 25-hydroxyvitamin D.

**Table 2:** Total serum calcium levels at baseline and after 12 weeks

	*Vitamin D insufficient group*		*Vitamin D sufficient group*	
	*Serum calcium at baseline (mg/dl)*	*Serum calcium at 12 weeks (mg/dl)*	*Serum calcium at baseline (mg/dl)*	*Serum calcium at 12 weeks (mg/dl)*
*N*	70	69	87	77
Mean±s.d.	9.0±0.8	9.4±0.6	9.5±0.6	9.3±0.5
Median	9.1	9.3	9.4	9.3
Range	6.6–10.3	8.2–12.6	8.1–11.2	7.9–10.5
>10.1 mg/dl	1	6	7	3
